# Comparative analysis of oral treponemes associated with periodontal health and disease

**DOI:** 10.1186/1471-2334-13-174

**Published:** 2013-04-11

**Authors:** Meng You, Sisu Mo, W Keung Leung, Rory M Watt

**Affiliations:** 1 Oral Biosciences, Faculty of Dentistry, The University of Hong Kong, Prince Philip Dental Hospital, 34 Hospital Road, Sai Ying Pun, Hong Kong; 2 Oral Diagnosis and Polyclinics, Faculty of Dentistry, The University of Hong Kong, Prince Philip Dental Hospital, 34 Hospital Road, Sai Ying Pun, Hong Kong; 3 Present Address: Department of Oral Radiology and State Key Laboratory of Oral Diseases, West China College of Stomatology, Sichuan University, Chengdu 610041, China

**Keywords:** Oral treponeme, *Treponema*, Periodontitis, Dentistry, Bacterial phylogeny, Phylogroup, Operational Taxonomic Unit, Bacterial etiology, Oral microbiota, Clinical study

## Abstract

**Background:**

Periodontal diseases, such as periodontitis, are chronic inflammatory infections affecting the gingivae (gums), underlying connective tissues and bone that support the teeth. Oral treponemes (genus *Treponema*) are widely-considered to play important roles in periodontal disease etiology and pathogenesis; however, precise relationships remain to be fully established.

**Methods:**

A 16S rRNA clone library-based approach was used to comprehensively characterize and compare the diversity of treponeme taxa present in subgingival plaque sampled from periodontitis patients (n = 10) versus periodontitis-free controls (n = 10). 16S rRNA gene sequences were assigned to operational taxonomic units (OTUs) using a 99% identity cut-off A variety of taxonomy (OTU) and phylogeny-based statistical approaches were used to compare populations of treponeme OTUs present in both subject groups.

**Results:**

A total of 615 plasmid clones containing ca. 1500 bp *Treponema* 16S rRNA gene sequences were obtained; 365 from periodontitis subjects, 250 from periodontitis-free controls. These were assigned to 110 treponeme OTUs. 93 OTUs were detected in the periodontitis subjects (mean 9.3 ± 5.2 OTUs per subject; range 9–26), and 43 OTUs were detected in controls (mean 4.3 ± 5.9 OTUs per subject; range 3–20). OTUs belonging to oral treponeme phylogroups 1–7 were detected in both subject sets. Phylogroup 1 treponemes had the highest levels of OTU richness (diversity) and clonal abundance within both subject groups. Levels of OTU richness and clonal abundance of phylogroup 2 treponemes were significantly higher in the periodontitis subjects (Mann Whitney *U*-test, *p* < 0.001). Both OTU-based and phylogeny-based analyses clearly indicated that there were significant differences in the composition of treponeme communities present in periodontitis versus control subjects. The detection frequency of five OTUs showed a statistically-significant correlation with disease status. The OTU (8P47) that corresponded to the type strain of *Treponema denticola* had the strongest association with periodontitis (*p* < 0.01).

**Conclusions:**

Higher levels of treponeme taxon richness and clonal abundance were associated with periodontitis. However, our results clearly indicated that subjects free from clinical symptoms of periodontal disease also contained highly diverse populations of treponeme bacteria within their subgingival microbiota. Our data supports the hypothesis that specific treponeme taxa are associated with periodontal disease.

## Background

Periodontal disease encompasses a range of chronic inflammatory infections that affect the gingiva (gums) and underlying connective tissues and bone that surround and support teeth [1-3]. It is the leading cause of tooth loss in adults over the age of 35, and it has been estimated that up to 90% of the global adult population may have at least a minor form of periodontal disease [1,3]. Periodontal disease, as typified by periodontitis, has a varied and highly complex polymicrobial etiology [4,5]. Subgingival (below the gum-line) plaque within diseased periodontal sites is typically enriched in anaerobic and proteolytic species of bacteria [6,7]. Investigations utilizing microscopy, culture-based and molecular-based approaches have previously revealed that oral spirochete bacteria are often highly-abundant within these ‘periodontal pockets’ of infection [6-12]. Molecular identification techniques based on the sequence of the highly-conserved 16S ribosomal RNA (rRNA) gene have demonstrated that all resident taxa of oral spirochete bacteria belong to the genus *Treponema*[13-15]. Studies have previously established that there is a positive relationship between the occurrence and severity of periodontitis, and the abundance of oral treponeme bacteria present in subgingival plaque within diseased sites [8,10-12,16,17]. *Treponema denticola* is the best characterized and most highly-studied species, and is considered a putative periodontal pathogen (periodontopathogen) [6,18-22]. However, despite extensive evidence of association, the precise etiological contribution of distinct oral treponeme ‘species’ towards periodontal disease causation or pathogenesis remains to be accurately established. This is exacerbated by the fact that the detailed composition of treponeme communities present within healthy subjects has been poorly studied.

Molecular analyses of treponeme populations in subgingival plaque samples using ‘spirochete specific’ 16S rRNA gene PCR primer sets have previously revealed a significant diversity of species and as-yet uncultivated species-level phylotypes [7,13,14,23]. Dewhirst *et al.* proposed a taxonomic framework for the systematic classification of human oral treponeme bacteria, which has become widely accepted [13,15]. They defined a phylotype as a set of near full-length 16S rRNA gene sequences (ca. 1500 bp) sharing >99% identity. Oral treponeme phylotypes are clustered into 10 distinct ‘phylogroups’, which share at least 90% 16S rRNA gene sequence identity. Recent estimates suggest there are ca. 50 treponeme phylotypes, which are also referred to as Operational Taxonomic Units (OTUs), present in the human oral cavity (based on a 98.5% 16S rRNA sequence identity cut-off) [14].

Thus far, 10 species of treponemes have been isolated and characterized within 7 of the 10 defined oral phylogroups: ‘*Treponema vincentii*’ [24,25] and *Treponema medium*[26] (phylogroup 1); *Treponema denticola*[27] and *Treponema putidum*[28] (phylogroup 2); *Treponema maltophilum*[29] and *Treponema lecithinolyticum*[30] (phylogroup 4); *Treponema amylovorum*[31] (phylogoup 5); *Treponema socranskii* (subspecies: *socranskii*, *paredis*, *buccale*, and ‘*04*’) [32,33] (phylogoup 6); *Treponema parvum*[34] (phylogoup 7); and *Treponema pectinovorum*[35] (phylogroup 8). Species belonging to phylogroups 3, 9 and 10 have yet to be cultivated. Although *Treponema pallidum*, *Treponema pedis* and *Treponema phagedenis* are genetically-related to *T. denticola* and *T. putidum* (phylogroup 2), they are not classified using the oral treponeme phylogroup taxonomy, as they are not considered to be resident species in the human oral cavity [15,36,37]. However, it should be noted that *T. pallidum* may be considered an infectious agent in the oral cavity [38].

To gain a more holistic understanding of the treponeme species (OTUs) associated with periodontal health and disease, we used a 16S rRNA gene clone library-based approach to comprehensively analyze treponeme populations present within pooled subgingival plaque samples from adult subjects with periodontitis (n = 10), as well as adult control subjects (n = 10) who were free from clinical symptoms of periodontitis. We found that both subject groups had diverse treponeme communities present in their subgingival plaque, which comprised many different OTUs. Clone enumeration indicated that certain treponeme OTUs were associated with periodontitis, whilst others were associated with disease-free status. Taken together, our results suggest that there is a complex relationship between treponeme communities and periodontal health status, and do not exclude the possibility that there may be certain OTUs (taxa) with increased pathogenic potential.

## Methods

### Clinical evaluation of periodontal health status and subgingival plaque sampling

Ethical approval was granted by the Institutional Review Board of the University of Hong Kong/Hospital Authority, Hong Kong West Cluster (UW 11–154). All subjects were recruited with informed consent, and this study was carried out in compliance with the Declaration of Helsinki. Subjects consented to their individual data being published. Inclusion/exclusion criteria; clinical evaluation of periodontal health status and subgingival plaque sampling methods for this subject group have previously been reported [39]. Briefly, standardized clinical parameters were evaluated for each subject: number of standing teeth; full mouth bleeding on probing (BOP) scores; pocket probing depths (PPD); radiographic evidence of bone loss; and number of sites with clinical attachment loss (CAL) ≥4 mm. After the careful removal of supragingival plaque, pooled subgingival plaque samples were collected from each subject: from all periodontal pockets ≥5 mm within the periodontitis subjects (n = 10), or from all asymptomatic sulci within the periodontitis-free control subjects (n = 10); using sterile Gracey or universal curettes, respectively. DNA was purified from the washed (PBS buffer) subgingival plaque samples within 30 min of collection, using a Wizard Genomic DNA Purification Kit, Promega; manufacturer’s protocol for gram-negative species).

### PCR amplification, cloning and analysis of 16S rRNA gene sequences

*Spirochaetes* and *Synergistetes* 16S rRNA sequences were selectively amplified from each purified subgingival plaque DNA sample (n = 20) using the TPU1 (5^′^-AGAGTTTGATCMTGGCTCAG-3^′^) and C90 (5^′^-GTTACGACTTCACCCTCCT-3^′^) primer set as previously reported [39]. Briefly, PCR amplicons (ca. 1500 bp in length; corresponding to positions 8–1503 of the *Escherichia coli* 16S rRNA gene) were gel purified (QIAquick Gel Extraction Kit, Qiagen), and ‘TOPO-cloned’ into pCR2.1 vectors (TOPO TA Cloning Kit, Invitogen). Ligation mixtures (n = 20) were transformed into *Escherichia coli* DH10B, plated onto Luria-Bertani (LB) 1% agar plates supplemented with kanamycin (50 μg/ml) and X-gal (5-bromo-4-chloro-indolyl-β-D-galactopyranoside, 20 μg/ml), then incubated overnight at 37 °C. Plasmid DNA was purified from 50–60 (white) colonies from each transformant plate (QIAprep Spin Miniprep Kits, QIAgen). 45–60 plasmids from each subject (n = 20) were sequenced bidirectionally using M13 forward and reverse primers [Beijing Genome Institute (BGI-Hong Kong Co. Ltd), Tai Po, Hong Kong].

16S rRNA gene sequences were assembled and trimmed using CodonCode Aligner 3.7.1 (Codon Code Corporation, Dedham, MA); then aligned using the Mothur software package [40], using the 16S ribosomal SILVA bacteria dataset [41] as a template, which was manually corrected prior to downstream analysis. 24 chimeric sequences were identified and removed using Chimera Slayer (Mothur). Sequences were taxonomically-classified using RDP Classifier to at least the level of phylum [42]. 16S rRNA sequences that corresponded to *Spirochaetes* were selected for further analysis.

A distance matrix was generated and sequences were assigned to OTUs at cut-off of 99% using the furthest neighbour algorithm in Mothur [40,43]. Single 16S rRNA gene sequences having the smallest total distance to all other sequences within each OTU were selected as representative sequences. A cut-off value of <98.5% identity to any sequence in the NCBI GenBank or Human Oral Microbiome Database (HOMD) [44] was used to identify novel OTUs.

### Phylogenetic relationships

A multiple sequence alignment comprising representative 16S rRNA gene sequences from each identified treponeme OTU was used as an input to examine phylogenetic relationships within the dataset. Jmodeltest v0.1.1 was used to determine the appropriate DNA substitution model and gamma rate heterogeneity using the Akaike Information Criterion (AIC) [45]. The generated model was used in all subsequent analyses except the Neighbour Joining (NJ) tree. The NJ tree was constructed following an evolutionary distance analysis of aligned sequences corrected by the Jukes-Cantor substitution model using MEGA 5 [46] supported by 500 bootstrap replicates. Maximum likelihood (ML) trees were generated using GARLI 1.0 with support calculated from 100 bootstrap replicates. Bayesian analysis was performed using MrBayes version 3.1.2 [47] using 2 replicates of 1 million generations with 4 chains, sampling every 100 generations. Bayesian posterior probabilities (PP values) were calculated from the consensus of remaining sampled trees after excluding the first 10% of trees as burn-in. PP values ≥0.95 were considered to have strong support. The relative clonal abundance for each OTU was visualized as a heat-map, constructed using ggplot2 from the R statistical computing package (http://www.r-project.org/) [48].

### Treponeme community comparisons

OTU- and phylogeny-based approaches were used to analyze and compare the composition of treponeme communities present in the subjects’ subgingival plaque samples (n = 20). Treponeme OTU populations in each subjects’ samples were compared and visualized as dendrograms according to the Yue and Clayton theta structural diversity measure in the Mothur software package [40]. For phylogeny-based cluster comparisons, principal coordinate analysis (PCoA) plots were generated using the distance matrix calculated using the unweighted UniFrac algorithm, based on NJ phylogenetic trees constructed using Clearcut (Mothur) using all identified treponeme 16S rRNA sequences as an input (n = 615) [49]. The composition of treponeme communities present in the samples from the periodontitis (n = 10) and control (n = 10) groups were analyzed at both the genus (i.e. entire dataset) and phylogroup levels; using ∫ − libshuff [50,51], unweighted and weighted UniFrac analysis [49,52] and Parsimony p-tests [53]. The *p*-value threshold was set to 0.05.

### Statistical analysis

Non-parametric Mann–Whitney *U* tests were used for all statistical tests between the two cohorts. The *p*-value threshold was set to 0.05.

### Sequence deposition

16S rRNA gene sequences of representative clones for each OTU were deposited in the NCBI GeneBank database with the accession numbers: JQ654102 - JQ654211 (see Additional file 1).

## Results

### Sequence analysis of 16S rRNA genes present in subgingival plaque samples

**Figure 1:**
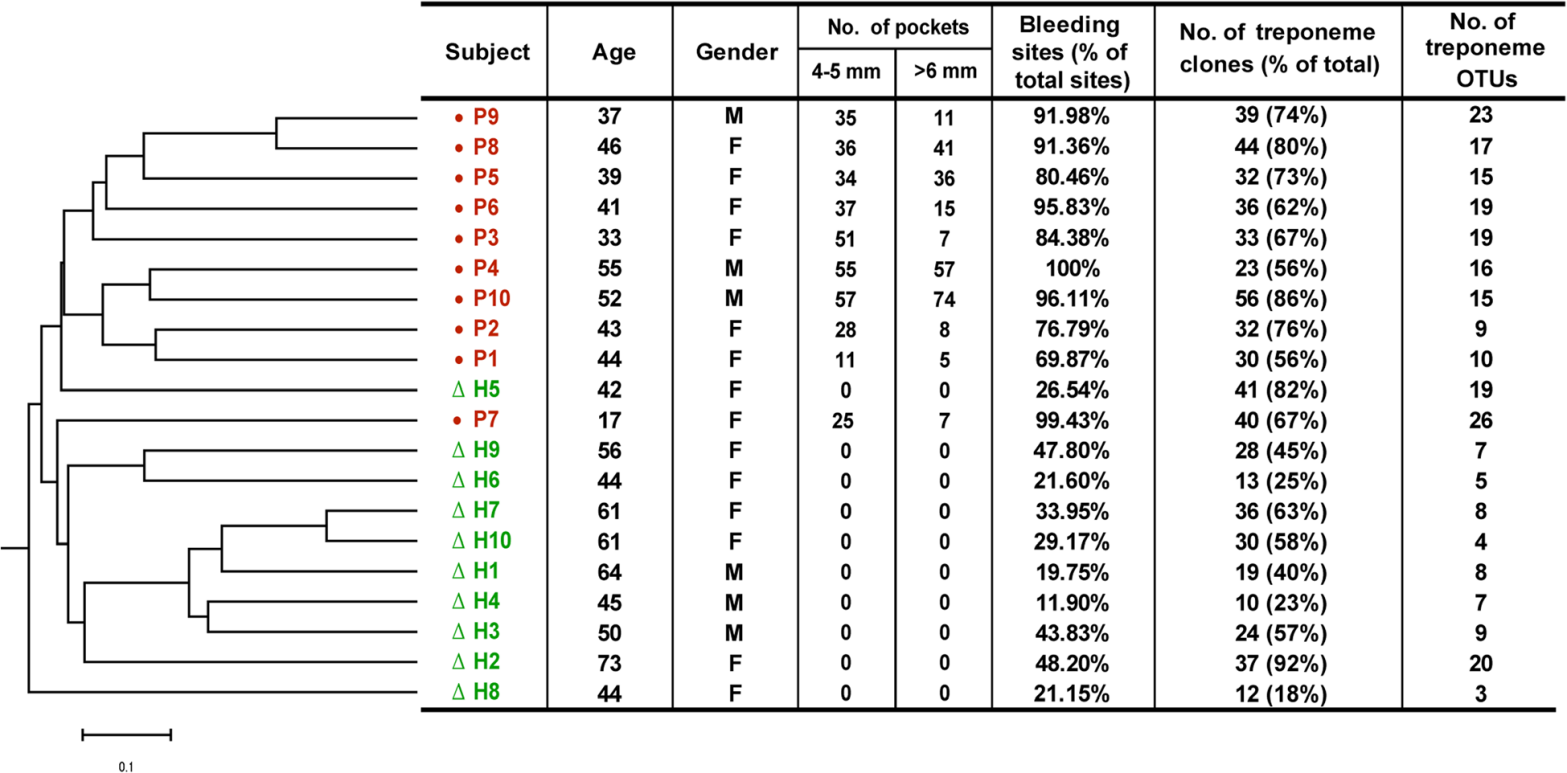
**Summary of the demography, periodontal status and composition of identified treponeme taxa, for each subject.** The dendrogram at the left hand side indicates the similarity of treponeme communities detected within each subject according to their OTU composition, as determined using the structure-based Yue & Clayton theta coefficient (Mothur software package).

A ‘spirochete-selective’ primer set was used to PCR amplify near full length 16S rRNA gene fragments from DNA purified from pooled (multi-site) subgingival plaque samples taken from 10 subjects with periodontitis (P1 – P10), as well as 10 controls free from clinical signs of periodontal disease (H1 – H10) [39]. A summary of the demographic details and clinical periodontal status for each of the subjects is shown in the table panel within Figure 1. Subgingival plaque samples were collected from a mean of 47.2 ± 31.2 (diseased) periodontal sites in the periodontitis group, and 165.0 ± 6.5 (disease-free) periodontal sites in the control group, as has previously been reported [39]. ‘TOPO’ plasmid libraries of PCR-amplified 16S rRNA clones were constructed, and inserts within ca. 50–65 plasmid clones from each subject were sequenced bidirectionally. After the removal of poor-quality or suspected chimeric sequences, a total of 1,030 16S rRNA sequences were obtained. There was no statistically-significant difference in the total number of 16S rRNA plasmid clones obtained from the periodontitis group (n = 520; range = 41–65) versus the control group (n = 510, range: = 40–65).

The bacterial origins of the cloned 16S rRNA genes were assigned using RDP classifier [42]. Results indicated that 70.2% (n = 365, range 23–56) of the plasmid clones from the periodontitis group, and 49.0% (n = 250, range 10–41) of the clones from the control group, contained 16S rRNA gene sequences that corresponded to members of the *Spirochaetes* phylum; all of which belonged to the genus *Treponema*. There was a statistically-significant difference in the number of treponeme plasmid clones obtained from each subject group (Mann Whitney *U* test; *p* < 0.05). The non-treponeme clones corresponded to members of the *Synergistetes* (n = 162), *Actinobacteria* (n = 167), *Fusobacteria* (n = 37), *Firmicutes* (n = 25) and *Proteobacteria* (n = 24) phyla; and have been described elsewhere [39].

### Assignment of treponeme 16S rRNA sequences to OTUs

Of the 615 treponeme 16S rRNA gene sequences identified, 521 (84.7%) were unique. These were assigned to 110 different treponeme OTUs (phylotypes) using a 99% sequence similarity cut-off (Additional file 1). The names of the OTUs reflected the clinical origin of the representative sequence (e.g. OTU 3H21 corresponded to clone 21 obtained from ‘healthy’ subject H3). However, it should be noted that this nomenclature system does not imply that a specific OTU is found exclusively in health or disease; it merely indicates the subject from which it was first detected. A total of 93 treponeme OTUs were detected within the periodontitis subjects (mean 9.3 ± 5.2 OTUs per subject; range 9–26), whilst 43 were detected within the control set (mean 4.3 ± 5.9; range 3–20). Only 26 of the 110 treponeme OTUs were common to both subject groups. Mann–Whitney U tests indicated that there was a statistically significant difference in OTU richness (i.e. numbers of different OTUs) found in the periodontitis and periodontitis-free groups (*p* < 0.01). Comparison with the 16S rRNA gene sequences in the NCBI GenBank and HOMD databases revealed that 22.7% (25/110) of the treponeme OTUs identified here were novel, based on a 98.5% sequence identity cut-off (Additional file 2). The percentage sequence similarity between the OTUs and their respective closest hits in the HOMD; namely the corresponding Human Oral Taxon (HOT) number, are also summarized in Additional file 1. 14 of the novel OTUs shared less than 97% sequence similarity to the respective closest match in the NCBI GenBank or HOMD databases. OTU 9P49 from phylogroup 2 and OTU 7P35 from phylogroup 5, were the most highly diverged, sharing less than 92% similarity with any previously identified 16S rRNA sequence.

### Comparisons of treponeme OTU richness, clonal abundance and phylogroup composition between the periodontitis and control groups

**Figure 2:**
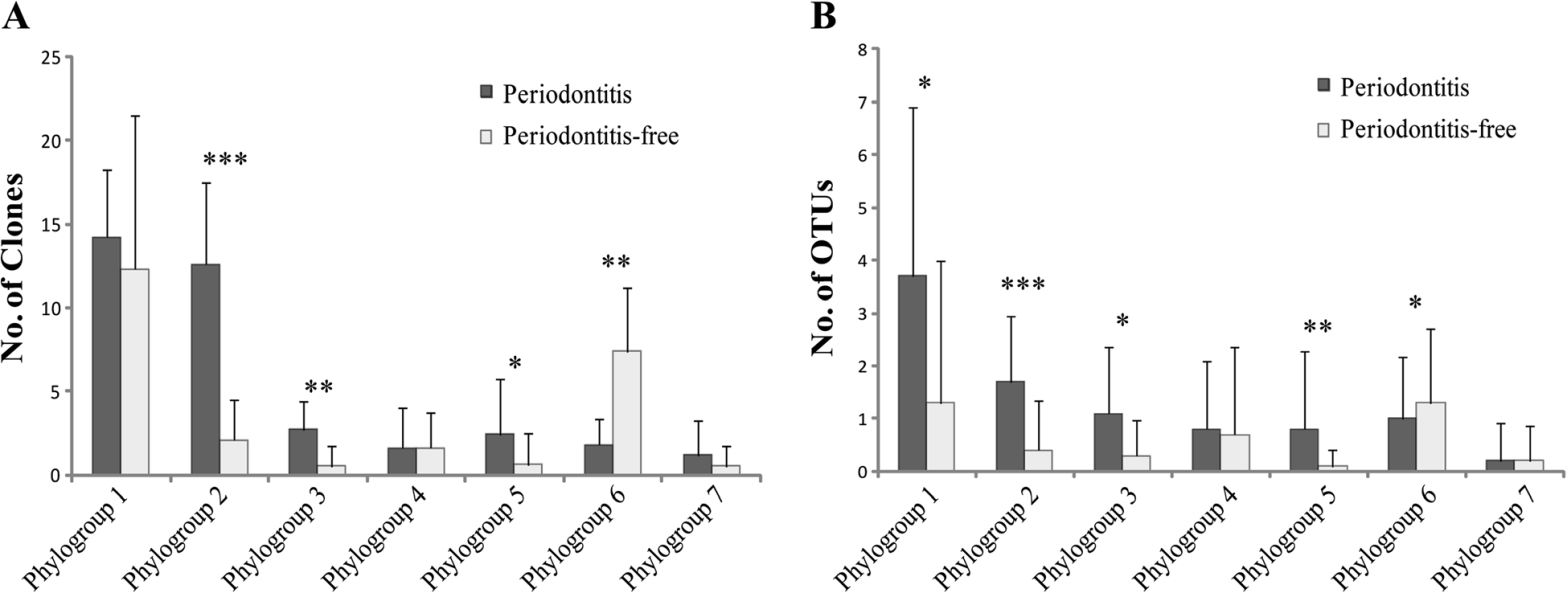
**Differences in treponeme phylogroup composition and detection frequency within the periodontitis and periodontitis-free subject groups.** Panel **A**. Plot showing the mean clonal abundance of treponeme taxa corresponding to oral treponeme phylogroups 1–7, within the periodontitis (n = 10, shaded dark gray) and periodontitis-free (n = 10, shaded light gray) subject groups. Y-axis: mean number of TOPO plasmids obtained per subject, whose cloned 16S rRNA inserts corresponded to taxa belonging to the respective oral treponeme phylogroups. X-axis: Phylogroups 1–7 = oral treponeme phylogroups 1–7. Panel **B**. Plot showing the mean number of OTUs detected within each oral treponeme phylogroup (Y-axis), in the periodontitis and periodontitis-free subject groups. Mean values are plotted with error bars indicating standard deviation. Significance: * = *p* < 0.05, ** = *p* < 0.01, *** = *p* < 0.001).

The 110 OTUs identified here could be assigned to seven of the ten oral treponeme ‘phylogroups’ previously defined by Dewhirst *et al.*[13]. Results are summarized in Figure 2 and Additional file 1. Clonal abundance was used as a semi-quantitative indicator of the abundance of each treponeme taxon within the respective subjects. This was defined as the number of ‘TOPO’ plasmids obtained that contained a cloned 16S rRNA gene corresponding to the respective OTU or phylogroup. Considering the entire dataset as a whole, phylogroup 1 contained both the largest proportion of plasmid clones (265/615; 43.1% of total), and the largest number of OTUs (41/110; 37.3% of total), compared to the other six phylogroups. Whilst there was no statistically-significant difference in the clonal abundance of phylogroup 1 sequences between the two subject groups, the periodontitis cohort contained higher levels of OTU richness within phylogroup 1 (*p* < 0.05, Mann–Whitney *U* test; see Figure 2). Most notably, there were a significantly higher number of phylogroup 2 clones within the periodontitis group, compared to the control group (*p* < 0.001). Phylogroup 2 clones constituted 34.5% (126/365) of the total plasmid count for the periodontitis group, whilst they comprised only 8.4% (21/250) of plasmids obtained from the periodontitis-free group. The periodontitis subjects also yielded significantly higher levels of OTU richness for phylogroup 2, compared with the controls (*p* < 0.001). There were analogous disease correlations for oral treponemes belonging to phylogroups 3 and 5, where both OTU richness and clonal abundance were significantly elevated in the periodontitis group, compared to the respective controls (*p* < 0.05; see Figure 2). In marked contrast, both the clonal abundance and levels of OTU richness within phylogroup 6 were significantly lower in the periodontitis group, compared to the periodontitis-free group (*p* < 0.05).

We also calculated the Chao1 and ACE estimates of taxon richness; the Shannon, and Simpson’s estimators of taxon diversity; as well as Good’s coverage; for the datasets obtained from the 20 subjects, at both the genus and phylogroup levels. Results are summarized in Additional file 3. The Chao 1 and ACE estimators both indicated that we had identified approximately half of the predicted number of treponeme OTUs present within the subgingival plaque of the 20 subjects. Good’s coverage estimator indicated that our relative sampling coverage was higher within phylogroups 1, 2, 6 and 7; and lower within phylogroups 3, 4 and 5.

### Comparisons of the composition of treponeme communities present in the periodontitis and healthy cohorts

**Figure 3:**
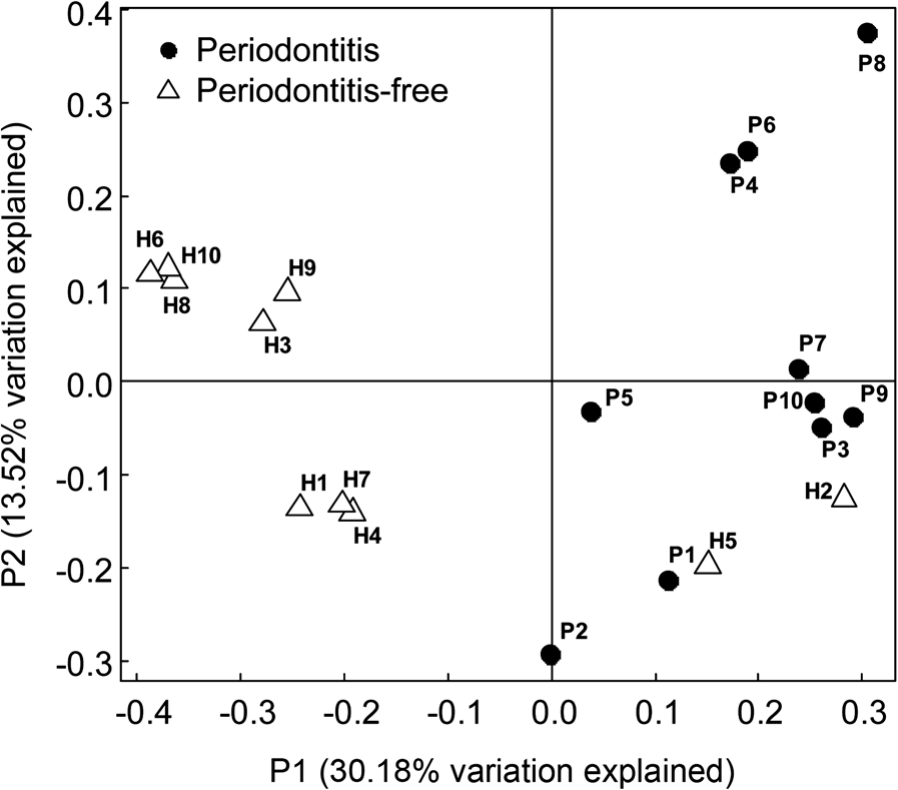
**Principal component analysis (PCoA) of variation amongst the treponeme communities present in each subject.** The PCoA was performed using a distance matrix calculated using an unweighted UniFrac algorithm based on a single neighbour-joining (NJ) tree containing all treponeme 16S rRNA gene sequences detected (n = 615). The PCoA scatter plot was constructed using the two principal axes (P1 and P2), which respectively explained 30.18% and 13.52% of the variation between treponeme communities. Black solid circles represent the 10 periodontitis subjects (P1-P10); unshaded triangles represent the 10 periodontitis-free subjects (H1-H10).

We systematically compared the treponeme communities present within each of twenty periodontitis and periodontitis-free subjects using two different types of cluster analysis: one that was OTU-based (i.e. taxonomy-based), and another that was phylogeny-based. The OTU-based Yue and Clayton (theta coefficient) measure of dissimilarity (Mothur software package) is a taxonomic approach that utilizes a binning procedure. In contrast, the phylogeny-based Unifrac analysis uses a Neighbour Joining (NJ) phylogenetic tree as an input, and the distance matrix is visualized using a principal component analysis (PCoA) scatter plot. These two approaches may be used to compare the taxonomic and genetic similarities between different communities, respectively. The dendrogram generated using the OTU-based Yue and Clayton theta coefficient is shown in Figure 1 (left panel). The subjects clustered according to their periodontal health status, with the periodontitis and control subjects being clearly-separated in the dendrogram; with the exception of only two subjects: H5 and P7. The clinical parameters determined for each subject are shown in a table alongside their reference code, age and gender; which clearly highlights the differences in gingival health between the two subject groups (Figure 1). It may be noted that subject P7 (age 17) was the only one affected with aggressive periodontitis, with the other 9 subjects having chronic periodontitis. The PCoA scatter plot of differences between the treponeme communities found within the subgingival plaque from each subject is shown in Figure 3. This was performed using a distance matrix calculated using an unweighted UniFrac algorithm, based on a single NJ tree comprising all 615 treponeme 16S rRNA sequences identified. The principal coordinates (axes) P1 and P2 were found to explain 30.18% and 13.52% of the data variation, respectively. Control subjects were generally well-separated from the periodontitis subjects in the plot (especially by P1), with the exception of H2 and H5. These results showed that the two subject groups were well-separated using both the treponeme OTU- and phylogeny-based cluster analysis methods. It should be noted however, that the relatively long branch-lengths in the OTU-based dendrogram (Figure 1) and the dispersed nature of subjects belonging to the same clinical group in the PCoA scatter plot (Figure 3), both indicate that the composition of the treponeme communities present within the individual subjects (in both subject groups) were highly variable.

In order to determine whether the separation of the periodontitis and control groups observed in the clustering analysis described above was statistically significant, we performed four different types of phylogeny-based pairwise comparisons. These included ∫ − libshuff, unweighted (UW) and weighted (W) UniFrac analyses, and Parsimony p-test algorithms. A single NJ phylogenetic tree containing all 615 treponeme 16S rRNA sequences (identical to that used for the PCoA analysis), was used as the input for all four methods. Results obtained are summarized in Table 1. All four approaches clearly indicated that the communities of treponemes present in the two subject sets had very distinct compositions, which were supported with high levels of statistical significance (*p* < 0.001).

**Table 1:** Treponeme community comparisons between the periodontitis and control groups at the phylogroup level

**Treponeme Phylogroup**	**1**	**2**	**3**	**4**	**5**	**6**	**7**	**All**
∫ − libshuff	***	***	*	*	***	*	ns	***
UW UniFrac	***	***	ns	ns	**	**	ns	***
W UniFrac	***	***	***	***	***	***	***	***
Parsimony p-test	***	***	*	ns	***	**	ns	***

Differences in the composition of treponeme OTUs belonging to phylogroups 1–7 that were detected in the periodontitis and periodontitis-free subject groups were determined using ∫ − libshuff, unweighted UniFrac, weighted UniFrac and parsimony p-test approaches. *UW UniFrac* = unweighted UniFrac; *W UniFrac* = weighted UniFrac. Significance: ns = *p* > 0.05; * = *p* < 0.05; ** = *p* < 0.01; *** = *p* < 0.001; *ns* = not significant.

### Comparisons between subgingival treponeme communities in the periodontitis and control groups at the phylogroup level

Analogous sets of hypothesis tests were performed to determine whether the treponeme communities within the subgingival plaque sampled from the periodontitis and control groups had similar structures at the phylogroup level. This was done to determine whether the differences in treponeme communities between the two subject groups were due to changes in the respective compositions of treponemes within all seven phylogroups, or predominantly due to changes within only one or a few phylogroups. As may be seen in Table 1, results from ∫ − libshuff, unweighted and weighted UniFrac analyses, and Parsimony p-tests all indicated that the structures of the treponeme communities corresponding to phylogroups 1, 2, 5 and 6 were significantly different in the periodontitis and control groups. All hypothesis tests, except for the unweighted UniFrac analysis, indicated that the composition of phylogroup 3 treponemes was significantly different in the disease versus control sets. However, corresponding results were ambiguous for the phylogroup 4 and 7 treponemes. The differences between the results obtained using the weighted and unweighted UniFrac methods most probably reflect the fact that the composition of the phylogroup 3, 4 and 7 treponeme communities present within the two subject groups were relatively similar; however, their respective clonal abundances in the 20 subjects were quite variable.

### Relationships between treponeme OTU phylogeny, clonal abundance and periodontal health status

**Figure 4:**
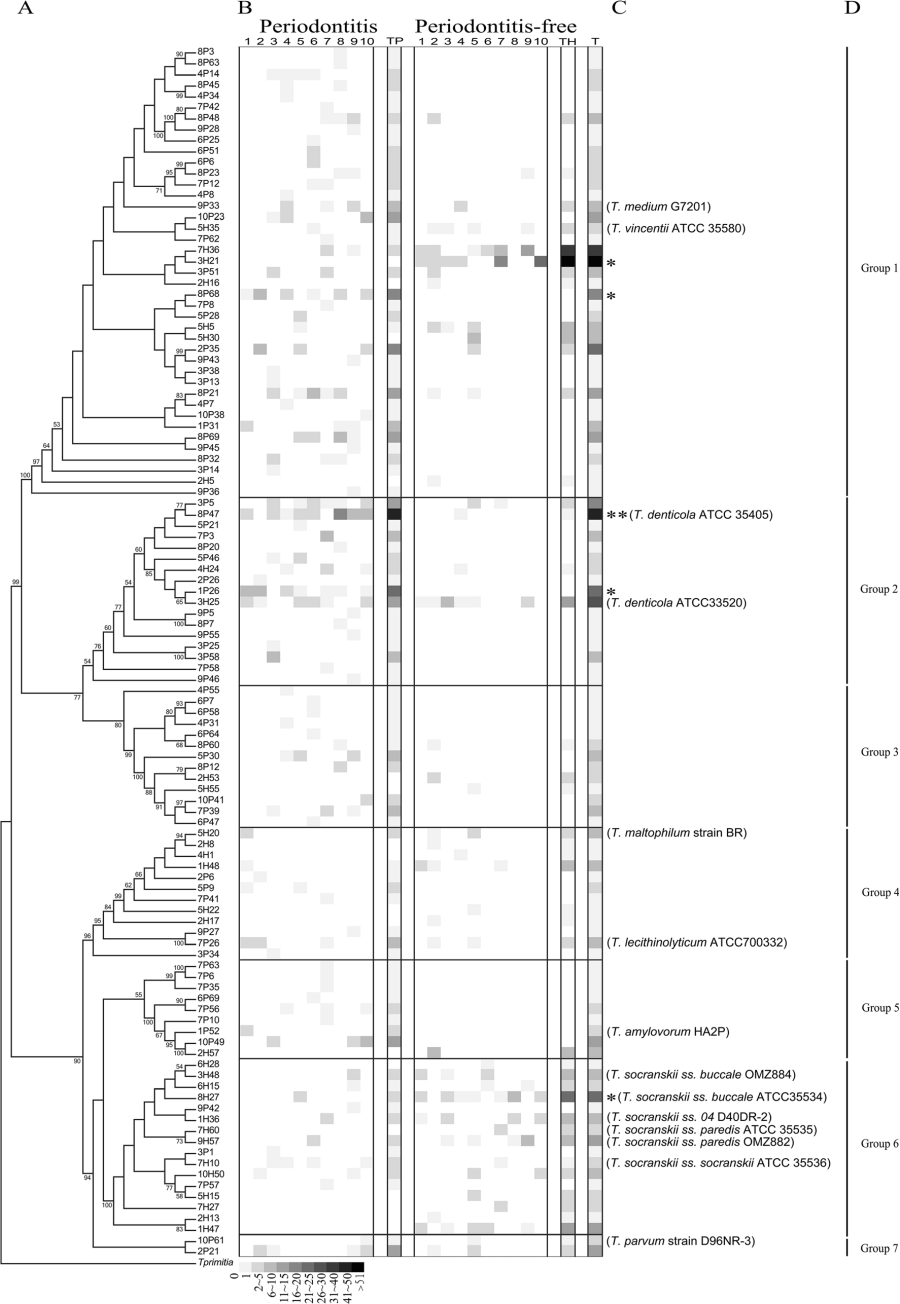
**Phylogenetic relationships and clonal abundance of the 110 Treponeme OTUs identified in this study.** Figure components (from left to right). (**A**) 16S rRNA gene phylogenetic tree of the 110 identified treponeme OTUs and one outgroup species (*Treponema primitia ZAS2*) shown in an ultrametric form. The tree was constructed using a Neighbour-joining (NJ) method with 500 bootstrap replicates. Bootstrap values ≥50 are shown at branch points. (**B**) Clonal abundance of each treponeme OTU (i.e. number of 16S rRNA plasmid clones obtained from each subject that correspond to an individual OTU), represented using grey-scale shaded boxes. Scale values are shown at the base of the figure. **1**–**10**: subject number in each cohort; **TP**: total clonal abundance of OTUs obtained from the periodontitis group; **TH**: total clonal abundance of OTUs obtained from the periodontitis-free control group; **T**: total clonal abundance of OTUs obtained from both subject groups; (**C**) Statistically-significant differences in the clonal abundance of respective OTUs detected within the periodontitis and periodontitis-free subject groups (Mann–Whitney *U* test, * = *p* < 0.05, ** = *p* < 0.01), Strain names in parentheses are representative reference strains belonging to the respective OTUs. (**D**) Group 1 – Group 7 = oral treponeme phylogroups 1–7.

Phylogenetic relationships between the 110 treponeme OTUs detected in the entire subject dataset were analyzed using Neighbour Joining (NJ), Maximum Likelihood (ML) and Bayesian (BA) approaches. The topologies of the dendrograms generated using these three methods were highly congruent with one another (data not shown). A NJ phylogram (i.e. NJ tree with branch lengths proportional to genetic distances) containing all 110 treponeme OTUs is shown in Additional file 4. The corresponding NJ cladogram (i.e. NJ tree shown in an ultrametric form, where branch lengths are not proportional to genetic distances) is shown in Figure 4, Panel A. The 16S rRNA gene from *Treponema primitia* ZAS-2 (a termite hindgut symbiont) was used as an out-group, to root the NJ tree. The 110 treponeme OTUs were well-separated into several distinct clusters supported with high bootstrap support (BS) values. The tree topology correlated closely with the taxonomic framework of oral treponeme phylogroups proposed by Dewhirst *et al.*[13]. The clonal abundance and distribution of each treponeme OTU within the pooled subgingival plaque samples collected from each of the 20 subjects is represented with a (grayscale) heat-map aligned to the right hand side of the NJ tree shown in Figure 4, Panel B. The combined clonal abundances of each OTU within the periodontitis (P) and periodontitis-free (H; ‘healthy’ control) subject groups are respectively indicated in the two columns labeled (TP) and (TH). The overall OTU clonal abundance (for both subject groups) is indicated in the column labeled (T). Reference strains of oral treponeme species that correspond to the treponeme OTUs detected in our cohort are shown to the right of the heat map (Panel C).

It may be seen in Panel C that the overall clonal abundance of OTUs corresponding to phylogroup 1 oral treponemes were fairly similar in both the periodontitis and control groups. However, the clones from the periodontitis group were widely distributed between numerous different OTUs, whilst those in the control set were concentrated within a relatively small number of OTUs. This is consistent with the overall differences in clonal abundance and OTU diversity shown in Figure 2. In particular, the heat map clearly illustrates that the periodontitis group contained a substantially higher diversity and clonal abundance of phylogroup 2 treponemes than the control group. The converse situation was found for phylogroup 6 treponemes, where both the OTU diversity and clonal abundance were higher in the periodontitis-free subject group. OTUs corresponding to treponeme phylogroups 3 and 5 treponemes were rarely detected in the control subjects.

Further focusing the analysis to specific treponeme OTUs, the detection frequency of five OTUs showed a statistically-significant correlation with disease status. OTU 8P68 from phylogroup 1, as well as OTUs 8P47 and 1P26 from phylogroup 2, were found more frequently in the periodontitis subjects. Most notably, the increased detection frequency of OTU 8P47 within the periodontitis subjects, which includes the ATCC 35405 type strain of *T. denticola*, had the highest levels of statistical support (*p* < 0.01, Mann–Whitney *U* test). In contrast, OTU 3H21 from phylogroup 1 and OTU 8H27 from phylogroup 6 were found more frequently in control subjects. It should be noted that OTU 8H27 contains the type strain for *T. socranskii* subspecies *buccale* (ATCC 35534).

## Discussion

In this study, we used a 16S rRNA clone library-based approach to systematically analyze and compare the diversity of treponeme bacteria OTUs (phylotypes) present within subgingival sites in Chinese subjects with periodontitis (n = 10), versus Chinese subjects free from clinical signs of periodontal disease (n = 10). Our results indicated that subgingival plaque samples collected from the diseased ‘periodontal pockets’ from periodontitis subjects contained treponeme communities that were significantly more diverse and more abundant than corresponding samples taken from healthy or mild gingivitis sites, from periodontitis-free subjects. OTU and phylogeny-based clustering methods both demonstrated that the periodontitis and control subjects contained quite distinct treponeme communities within their periodontal niches. Most notably, there were significantly higher levels of OTU richness and clonal abundance of phylogroup 2 oral treponemes present in the periodontitis subjects.

Although treponeme taxa are generally considered to play an etiological role in periodontal disease [10,11], their clinical distributions have been relatively poorly studied at the molecular level. To the best of our knowledge, Dewhirst *et al.*[13] and Paster *et al.*[7] appear to have published the only in-depth 16S rRNA clone-based analyses of oral treponeme populations associated with periodontal health and disease. However, in these reports, the relative numbers of 16S rRNA gene sequences analyzed from periodontally-diseased subjects were several-fold higher than those from ‘healthy’ control subjects; making an objective comparison problematic. Here, we directly compared differences in treponeme OTU composition within periodontitis and periodontitis-free subjects using a similar depth of sampling. As such, this study also represents a detailed molecular survey of oral treponeme diversity within subjects with no clinical signs of periodontal disease. By obtaining and analyzing more than 250 near full-length treponeme 16S rRNA sequences from periodontitis-free subjects, our results clearly reveal that shallow gingival sulci contain diverse populations of treponeme bacteria. This is consistent with data obtained from previous analyses of supra- and subgingival plaque samples from ‘healthy’ individuals, using next-generation DNA sequencing approaches [54,55].

The 521 unique treponeme 16S rRNA gene sequences identified in the entire dataset were assigned to 110 different OTUs using a 99% similarity cut-off. We defined our treponeme OTUs using this relatively high threshold value, in order to achieve more effective taxonomic resolution, and more precise disease associations with specific OTUs. 29 of these OTUs were deemed novel (ca. 26% of total), based on a 98.5% sequence similarity cut-off to the 16S rRNA gene sequences in the NCBI GenBank database (with lengths >1,400 nt). Our results are comparable to those reported by Dewhirst *et al.* in their recent catalogue of the human ‘oral microbiome’. These investigators identified 73 treponeme taxa, which corresponded to 49 treponeme OTUs using a 98.5% identity cut-off [14]. It may be noted that the 110 treponeme OTUs defined here correspond to 75 OTUs if a 98.5% cut-off is used (data not shown). Our results are also consistent with those obtained from a pyrosequencing analysis of supragingival plaque from 98 individuals with good oral health, where 118 *Treponema* OTUs were identified [54].

It may be noted that the primer set employed here enables the analysis of taxa belonging to both the *Spirochaetes* and *Synergistetes* phyla with high levels of selectivity [39]. Thus, this approach may be used to simultaneously survey communities of two distinct groups of bacteria associated with periodontal infections [7,14,39]. Furthermore, the amplification of near full-length 16S rRNA genes facilitates accurate taxonomic assignment, which is especially important for the discrimination of closely-related treponeme species, subspecies and phylotypes (e.g. see below). However, clone-based approaches are relatively time-consuming, expensive and labour-intensive when applied to the analysis of large sample sets. In such cases, the use of next-generation sequencing technologies may offer practical advantages in terms of cost, throughput and depth of sampling.

All of the treponeme OTUs detected in this study belonged to oral phylogroups 1–7; with taxa belonging to these 7 phylogroups being identified in both the periodontitis and periodontitis-free subject sets. As may be seen in Additional file 4, three of the OTUs assigned to phylogroup 5 (7P6, 7P35, 7P63), formed a clade that was quite distinct from that containing the other 6 OTUs within this phylogroup. The taxonomic classification of these three OTUs within the oral treponeme phylogroup framework may be revisited at some point in the future, in light of additional sequence data which may become available within this genus. *T. pectinovorum* (phylogroup 8) was not detected in our cohort. This is consistent with the data previously reported by Paster and Dewhirst *et al.*, who similarly failed to detect *T. pectinovorum* within any of the 31 subjects studied [7,13]. Similarly, we did not identify treponeme taxa belonging to phylogroups 9 or 10. There are presently very few phylogroup 9 or 10 16S rRNA gene sequences in the publically-available databases. These have been predominantly detected in subjects with acute necrotizing ulcerative gingivitis (ANUG, also known as trench mouth); with each comprising a single OTU (phylotype) [7,13]. It appears likely that treponemes belonging to phylogroups 9 and 10 are either uncommon inhabitants of the human oral cavity, or are generally present in extremely low proportions.

Within both subject groups, as well as the combined subject dataset, oral phylogroup 1 treponemes were the most commonly detected (43.1% of total) and had the highest numbers of component OTUs (37.3% of total). This finding is in good agreement with data from previous investigations [7,13]. The two representative species from phylogroup 1: ‘*T. vincentii’* and *T. medium*, have both been implicated in the etiology of periodontal disease, due to their increased detection frequency at diseased sites [16,56,57]. Here, *T. vinc*entii and *T. medium* comprised only ca. 5% of the total number of clones obtained for oral phylogroup 1 treponemes; the rest corresponding to yet-to-be cultivated OTUs. This is consistent with the results of Möter *et al.*, who reported that the combined detection frequency for *T. vinc*entii and *T. medium* cells represented less than ca. 20% of that observed for phylogroup 1 treponemes as a whole [58]. Taken together, data strongly indicates that phylogroup 1 constitutes the largest, most highly populated and most diverse grouping of oral treponeme taxa. It is interesting to note that the two phylogroup 1 OTUs (8P68, 3H21) that we found to be associated with periodontitis subgingival plaque (*p* < 0.05), correspond to as-yet uncultivated treponeme taxa. Additional studies will be required to further elucidate their putative roles in periodontal disease etiology.

The clonal abundance and OTU diversity of phylogroup 2 treponemes were both significantly higher in the periodontitis subjects compared with periodontitis-free controls (*p* < 0.001). In the diseased cohort, they comprised 34.5% of plasmids obtained, corresponding to 17 OTUs; whilst in the control subjects, they accounted for only 8.4% of clones, which corresponded to only 4 OTUs. As such, phylogroup 2 treponemes appear to be the most reliable indicators of periodontal disease status. Furthermore, the OTU that corresponds to the ATCC 35405 type strain of *T. denticola* (OTU 8P47) was the taxon most strongly associated with periodontitis in our study (*p* < 0.01); constituting more than 30% of all phylogroup 2 clones detected in the subgingival plaque of periodontitis subjects. These results are in good agreement with a large body of results in the scientific literature, which have consistently linked *T. denticola* and phylogroup 2 treponemes with periodontitis, and other forms of periodontal disease [17-19,57,59-61].

*Treponema socranskii* comprises multiple subspecies (e.g. ss. *socranskii*, ‘*04’*, *paredis*, *buccale*), as well as several poorly-characterized ‘subspecies-level’ phylotypes (oral taxons) [7,13,33]. Here, we found that the taxon diversity and detection frequency of phylogroup 6 treponemes were both higher in the control group than in the disease set (Figure 2). One OTU (8H27), which corresponded to the type strain of *T. socranskii* subsp. *buccale* (ATCC 35534), was detected more frequently in the control subjects (*p* < 0.05). This initially appears to contradict several previous reports, where *T. socranskii* taxa have been associated with periodontal disease e.g. [16,22,62]. However, it should be noted that these previous investigations have investigated *T. socranskii* at the species level, or have employed lower levels of taxonomic discrimination than were used here. Upon closer inspection, data from the literature suggests that the various subspecies and phylotypes of *T. socranskii* may have differing associations with periodontal disease [7,63]. In one report, *T. socranskii* ss. *socranskii* and the *Treponema sp.* 6:G:G47 oral clone were detected more frequently in periodontally-diseased subjects than *T. socranskii* ss. *buccale*[63]. Discrepancies in disease associations also appear to reflect notable differences in the cohorts analyzed. *T. socranskii* taxa were found to be more abundant in subjects with ANUG, HIV-periodontitis or refractory periodontitis, rather than (chronic) periodontitis [7,13]. It also worth mentioning that results from a recent pyrosequencing-based investigation indicated that that *T. denticola* and two phylogroup 1 treponeme taxa were significantly associated with periodontitis, whilst *T. socranskii* taxa were not [17]. It is clear that additional detailed investigations within other subject cohorts are required to shed further light on this complex and controversial issue.

Whilst the majority of the OTUs detected in the subgingival plaque from periodontitis-free group (26/43 OTUs) were also present in the periodontitis set, their overall OTU compositions were notably distinct. This indicated there were major differences in overall treponeme community structures within the two subject groups. There were also considerable subject-to-subject variations in treponeme composition. This is perhaps unsurprising, as periodontal disease is a complex, multifactorial disease with a highly diverse microbial etiology [4,7,17]. Host physiology, as well as major differences in the biofilm microenvironments present within deeply-infected periodontal pockets versus shallow sulci, most-likely play major roles in modulating subgingival treponeme populations [64-66]. It remains to be seen whether the respective variations in treponeme OTU composition and relative abundance are correlated with specific changes in the overall subgingival microbial ecology; in particular species that may have syntrophic (nutritional) or inter-generic cell-binding relationships with treponemes, such as *Porphyromonas gingivalis* and *Fusobacterium nucleatum*[67,68].

## Conclusions

Here we show that diverse populations of treponeme species and uncultivated OTUs inhabit periodontal niches within periodontitis-free (‘healthy’) as well as periodontally-diseased subjects. Our data is consistent with the hypothesis that periodontitis is associated with significant changes in the composition of the community of treponeme OTUs present within the subgingival microbiota. We consider it likely that this may be accompanied with distinct population changes within other oral microbial taxa, as part of a dysbiosis in periodontal ecology. Our data also suggests that specific treponeme OTUs may play a more prominent role in the etiopathology of periodontal disease. Future detailed investigations within other patient cohorts will be required to clarify the putative involvement of these treponeme OTUs in periodontal disease processes.

## Abbreviations

OTU: Operational taxonomic unit; NJ: Neighbour joining; ML: Maximum likelihood; nt: Nucleotide; ATCC: American type culture collection; BOP: Bleeding on probing; PPD: Pocket probing depth; CAL: Clinical attachment loss; PBS: Phosphate buffered saline; PCoA: Principal component analysis; ANUG: Acute necrotizing ulcerative gingivitis.

## Competing interests

The authors declare that they have no competing interests; financial or otherwise.

## Authors’ contributions

Conceived the study: RMW, WKL. Designed and performed the practical experimental work: MY, SM, WKL, RMW. Designed and performed the computational and statistical analyses: MY, SM, RMW, WKL. Wrote the manuscript: MY, WKL, RMW. All authors read and approved the final manuscript.

## Pre-publication history

The pre-publication history for this paper can be accessed here:

http://www.biomedcentral.com/1471-2334/13/174/prepub

## Supplementary Material

Additional file 1 Treponeme OTUs identified in this study; including assigned names, accession numbers of representative sequences, and closest matches in the Human Oral Microbiome Database (HOMD). Click here for file

Additional file 2 Summary of novel treponeme OTUs identified in this study, including closest related sequences in the NCBI GenBank database. Click here for file

Additional file 3 Observed OTU numbers and estimates for OTU richness and diversity within each treponeme phylotype, for entire subject dataset. Click here for file

Additional file 4 **Neighbour-joining phylogram of the 110 treponeme OTUs identified in this study.** The tree is rooted with the 16S rRNA gene sequence of *Treponema primitia ZAS2.* The tree was constructed with 500 bootstrap replicates, with bootstrap values ≥50 shown at branch points. Scale bar: 0.02 substitutions per site. Click here for file
