# Supplementary figures and images for: Comparative analysis of oral treponemes associated with periodontal health and disease
Source: BMC Infect Dis. 2013 Apr 11;13:174. doi: 10.1186/1471-2334-13-174 (PMC3637317; doi:10.1186/1471-2334-13-174)

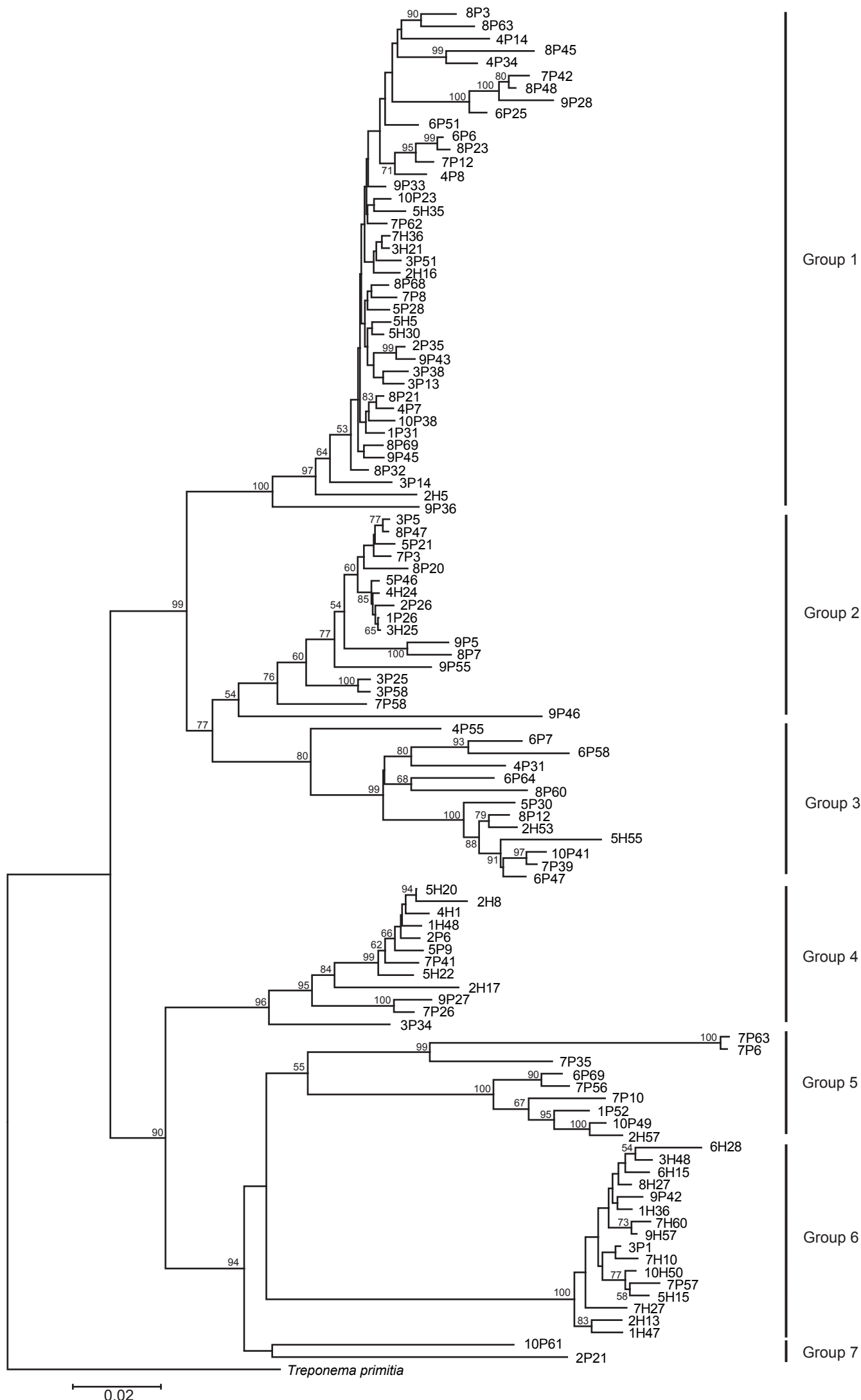

Supplement: Additional file 4 — Neighbour-joining phylogram of the 110 treponeme OTUs identified in this study. The tree is rooted with the 16S rRNA gene sequence of Treponema primitia ZAS2. The tree was constructed with 500 bootstrap replicates, with bootstrap values ≥50 shown at branch points. Scale bar: 0.02 substitutions per site. [file 1471-2334-13-174-S4.pdf]
